# Zika Virus Screening among Spanish Team Members After 2016 Rio de Janeiro, Brazil, Olympic Games

**DOI:** 10.3201/eid2308.170415

**Published:** 2017-08

**Authors:** Natalia Rodriguez-Valero, Alberto M. Borobia, Mar Lago, Maria Paz Sánchez-Seco, Fernando de Ory, Ana Vázquez, Jose Luis Pérez-Arellano, Cristina Carranza Rodríguez, Miguel J. Martínez, Alicia Capón, Elias Cañas, Joaquin Salas-Coronas, Arkaitz Azcune Galparsoro, Jose Muñoz

**Affiliations:** IS Global-Hospital Clínic de Barcelona, Barcelona, Spain (N. Rodriguez-Valero, J. Muñoz);; Hospital La Paz-Carlos III, Madrid, Spain (A.M. Borobia, M. Lago);; Instituto de Salud Carlos III, Madrid, Madrid, Spain (M.P. Sánchez-Seco, F. de Ory, A. Vázquez);; Complejo Hospitalario Universitario Insular Materno Infantil, Las Palmas de Gran Canaria, Spain (J.L. Pérez-Arellano, C. Carranza Rodríguez);; Hospital Clinic de Barcelona, Barcelona, Spain (M.J. Martínez, A. Capón);; Hospitales Universitarios Virgen del Rocío, Sevilla, Spain (E. Cañas);; Hospital de Poniente, El Ejido, Spain (J. Salas-Coronas);; Hospital Universitario de Donostia, Donostia, Spain (A. Azcune Galparsoro)

**Keywords:** Zika virus, viruses, Spain, Rio de Janeiro, Brazil, Olympic Games, Olympic team, screening, arboviruses, mass gathering event, vector-borne infections

## Abstract

We evaluated the risk for the Spanish Olympic Team acquiring Zika virus in Rio de Janeiro, Brazil, during 2016. We recruited 117 team members, and all tested negative for Zika virus. Lack of cases in this cohort supports the minimum risk estimates made before the Games.

The current Zika virus epidemic became a major concern for national Olympic delegations before they traveled to Rio de Janeiro, Brazil, during summer 2016. Fear about individual consequences of the infection, such as congenital or neurologic disorders, were common among athletes and other participants of the Olympic Games and led some persons not to attend the Games for these reasons. The possibility of the Olympics contributing to a global spread of the Zika virus epidemic also was a concern, initially raised by ≈100 academic researchers, expressed in an open letter addressed to the World Health Organization (WHO) in May 2016 (*1*,*2*).

The risk for Zika during the Rio de Janeiro Olympic Games was estimated to be very low in different models published in medical journals (9 × 10^−6^ to 3 × 10^−5^) (*3*–*6*). After considering these figures, WHO advised that the Games should not substantially affect the epidemic (*7*).

To evaluate the risk for the Spanish Olympic Team acquiring Zika virus, our research group from 6 hospitals in Spain invited members of the Spanish delegation to participate in a serologic study of Zika virus 20 and 30 days after returning from Rio de Janeiro. The study was conducted in 6 different recruiting Tropical Medicine Units in cities in Spain (Barcelona, Madrid, Sevilla, San Sebastian, Las Palmas de Gran Canaria, and Almeria).

Athletes and other participants were invited to participate in the study through the Spanish Olympic Committee. A total of 117 Olympics participants accepted and were included in the study during September and October 2016. After providing oral and written information, study participants signed an informed consent form, and demographic and health data were recorded in a medical questionnaire. A total of 10 mL of blood was drawn from each participant, and serologic tests for Zika virus (immunofluorescence antibody assay; EUROIMMUN, Luebeck, Germany), dengue virus (ELISA; Panbio, Kyonggi-do, Republic of Korea), and chikungunya virus (immunofluorescence assay; EUROIMMUN) were conducted at the Instituto de Salud Carlos III (Spanish National Reference Laboratory, Madrid, Spain). For all samples initially testing positive for Zika virus, we conducted microneutralization testing.

Twenty-one participants had >1 signs or symptoms while in Brazil: 18% rash, 23% fever (temperature >38°C), 14% itching, 9% of conjunctival hyperemia, 9% arthralgia, 14% myalgia, 40% malaise, 9% lymphadenopathy, 32% headache, and 19% gastrointestinal symptoms. Ninety-nine percent of participants received Zika virus counseling before they traveled to Rio de Janeiro, including the advice of having protected sex during and after the Games (Table).

**Table:** Demographic and travel-related characteristics of 117 Spanish athletes who attended the Olympic Games, Rio de Janeiro, Brazil, 2016*

Characteristic	Results
Sex	
M	76 (65.0)
F	41 (35.0)
Age, y, median ± SD	35.54 ± 9.46
Athletes	
All athletes	53 (45.3)
Outdoor athletes	35 (66.0)
Spanish nationality	112 (95.7)
Chronic disease	6 (5.1)
Immunosuppression	0
Current pregnancy, own or partner’s	9 (7.7)†
Intention to conceive within the following 6 mo	29 (24.8)
Sex	
M	22 (75.9)
F	7 (24.1)
Vaccination and travel advice	
Vaccine	
Yellow fever	23 (19.6)
Japanese encephalitis	0
Tickborne encephalitis	0
Attendance at a travel clinic	115 (98.3)
Zika advice included	116 (99.2)
>1 Visit to a tropical country	74 (63.3)
Previous diagnosis of dengue	0
During the trip	
Length of stay, d, median ± SD	21.35 ± 9.05
Places visited	
Rio de Janeiro	103 (88)
Rio de Janeiro, Deodoro, and Barra	7 (6.0)
Rio de Janeiro and Ilha Grande	4 (3.4)
Rio de Janeiro and Paraty	1 (0.85)
Rio de Janeiro and French Polynesia	1 (0.85)
Use of bed nets or air conditioners	61 (52.6)
Use of repellent	111 (94.9)
Risky sexual behavior	2 (1.7)
Recall >1 mosquito bite during stay	56 (47.9)

*All values are no. (%) unless otherwise indicated. †Male participants’ partners who were pregnant before the Games.

For 4 persons, test results for Zika virus IgG was positive; IgM and neutralization testing yielded negative results. The 4 Zika virus IgG–positive participants had received previous yellow fever vaccination and were asymptomatic. One sample showed Zika virus IgM in the absence of specific IgG; the results were confirmed in a follow-up sample. Thus, the sample was classified as false positive.

Study participants were advised to wait to conceive in accordance with WHO specifications: 6 months for men, 2 months for women. Participants with pregnant partners were advised to use condoms during the entire pregnancy.

A lack of Zika cases in this cohort supports the risk calculations made before the Games and the WHO statement that there were no Zika cases associated with the Olympic Games (*8*). Although 48% of participants in our study recalled at least 1 mosquito bite during the stay, the overall absence of cases in the Rio de Janeiro population during July and August 2016 (*9*,*10*) is believed to be due to the vector-control efforts by Brazilian authorities before the Games and to the winter weather, leading to a low presence of adult mosquitoes and mosquito bites (*5*,*6*).
